# Draft Genome Sequence of Streptomyces sp. Strain RKND-216, an Antibiotic Producer Isolated from Marine Sediment in Prince Edward Island, Canada

**DOI:** 10.1128/MRA.00870-19

**Published:** 2019-08-29

**Authors:** Libang Liang, Bradley A. Haltli, Russell G. Kerr

**Affiliations:** a Department of Chemistry, University of Prince Edward Island, Charlottetown, Prince Edward Island, Canada; b Department of Biomedical Sciences, Atlantic Veterinary College, University of Prince Edward Island, Charlottetown, Prince Edward Island, Canada; c Nautilus Biosciences Croda, Charlottetown, Prince Edward Island, Canada; University of Rochester School of Medicine and Dentistry

## Abstract

Streptomyces sp. strain RKND-216 was isolated from marine sediment collected in Prince Edward Island, Canada, and produces a putatively novel bioactive natural product with antitubercular activity. The genome assembly consists of two contigs covering 5.61 Mb. Genome annotation identified 4,618 predicted protein-coding sequences and 19 predicted natural product biosynthetic gene clusters.

## ANNOUNCEMENT

As part of our ongoing efforts to discover bioactive natural products from Streptomyces spp. (1), we identified *Streptomyces* sp. strain RKND-216 as a producer of a putatively novel antitubercular natural product. *Streptomyces* sp. RKND-216 was isolated from sediment collected off the coast of Burnt Point, Prince Edward Island, Canada (46.170339, −62.505634), at a depth of 13 m. The bacterium was isolated by dilution plating of 1 g of sediment on modified raffinose histidine agar (1% raffinose, 0.1% histidine, 0.1% KH_2_PO_4_, 0.0001% FeSO_4_·7H_2_O, 1.8% Instant Ocean, 1.2% noble agar [pH 7.7 ± 0.25]) supplemented with cycloheximide (50 mg liter^−1^), followed by incubation at room temperature (22°C to 25°C). Analysis of the *Streptomyces* sp. RKND-216 16S rRNA gene sequence indicated that the closest match in the GenBank 16S rRNA gene sequence database was Streptomyces qinglanensis (98.92% identity; GenBank accession number NR_109303). To facilitate analysis of biosynthetic gene clusters (BGCs) encoded in the genome of this organism, we obtained a draft whole-genome sequence.

*Streptomyces* sp. RKND-216 was cultured in 7 ml of marine International *Streptomyces* Project 2 (ISP2) broth (0.4% yeast extract, 1% malt extract, 0.4% dextrose, 1.8% Instant Ocean, 2% agar) at 30°C with shaking at 200 rpm. DNA was isolated from 1.8 ml of the culture using the DNeasy UltraClean microbial kit (Qiagen) following the manufacturer’s protocol, and 16 μg of high-quality genomic DNA was obtained. Library preparation and Pacific Biosciences RS II DNA sequencing were performed by McGill University and the Génome Québec Innovation Centre using a standard PacBio protocol for sheared large-insert (20-kb) libraries and a PacBio RS II single-molecule real-time (SMRT) platform with P4-C2 chemistry (Pacific Biosciences, USA). Sequencing was performed using one SMRT cell, which generated a total of 108,832 raw subreads (1,604,348,738 bp) with an average length of 14,741 bp, resulting in 285-fold coverage of the assembled genome sequence. The raw reads were filtered for quality using the SMRT Analysis software (version 2.3.0) (2) with a minimum subread length of 500, a minimum polymerase read quality of 0.75, and a minimum polymerase read length of 100. *De novo* genome assembly of the filtered reads was performed by the sequencing facility using the Hierarchical Genome Assembly Process (HGAP2)/Quiver protocol (2) (seed read length, 18,610; merylTheads, 7; ovlThreads, 1; merCompression 0; merSize, 14; merylMemory, 100,000; ovlErrorRate, 0.06; ovlMinLen, 40; frgMinLen, 500, ovlStoreMemory, 90,000; ovlConcurrency, 4; ovlCorrConcurrency, 4; cnsConsurrency, 7; and fraCorrThreads, 7). Assembly resulted in the generation of two contigs with lengths of 5,584,260 bp (72.6% G+C content, contig 1) and 30,487 bp (72.1% G+C content, contig 2). Circlator was used with default parameters (3), and it did not circularize either contig. Additionally, manual BLASTN analysis (4) did not identify internal direct overlapping regions when the ends of each contig were queried against the source contig. No overlapping regions were detected in contig 1 or contig 2. The predicted coding sequences of contig 2 were analyzed by BLASTP (5), and the majority of matches were to *Actinobacteria* genome sequences rather than plasmid-derived sequences. These data suggest that contig 2 is an unassembled component of the *Streptomyces* sp. RKND-216 chromosome.

The *Streptomyces* sp. RKND-216 draft genome was annotated with the Prokaryotic Genome Annotation Pipeline (PGAP version 4.8) (6), yielding 4,618 predicted protein-coding sequences, 59 tRNAs, and 18 rRNA genes (6 rRNA operons) from both contigs. AntiSMASH version 5.0.0 (7) was used to identify and annotate 19 putative BGCs from contig 1, which included one siderophore, one lanthipeptide, one linaridin, one ectoine, two terpene, four NPRS/NRPS-like, and nine hybrid BGCs. The AntiSMASH analysis identified four BGCs in contig 1 that shared a high percentage of genes with the following known BGCs: branched-chained fatty acids (100% similarity), SAL-2242 (100% similarity) (8), ectoine (100% similarity) (9), and mirubactin (78% similarity) (10). No BGCs were detected by AntiSMASH in contig 2. Characterization of BGCs identified in the RKND-216 genome will be described elsewhere.

### Data availability.

This whole-genome sequencing project has been deposited at DDBJ/ENA/GenBank under the accession number SSBQ00000000. Raw sequencing data sets, including the longest filtered subreads, filtered subreads, and circular consensus sequences (CCSs), have been registered in the NCBI SRA database under the accession number PRJNA529918.
